# Medication Adherence and Health‐Related Quality of Life Among Hypertensive Patients in Nepal: A Cross‐Sectional Study

**DOI:** 10.1002/puh2.70289

**Published:** 2026-06-01

**Authors:** Supuspa Lama, Prasanna Dahal, Sunil Neupane, Bipindra Pandey, Dharanidhar Baral, Kadir Alam, Sagun Kharel

**Affiliations:** ^1^ Department of Pharmacy Purbanchal University School of Health Sciences, Gothgaun Sundarharaicha Nepal; ^2^ Chettinad School of Pharmaceutical Sciences, Chettinad Hospital and Research Institute Chettinad Academy of Research and Education Kelambakkam Tamil Nadu India; ^3^ Department of Public Health Purbanchal University School of Health Sciences, Gothgaun Sundarharaicha Nepal; ^4^ Department of Pharmacy Madan Bhandari Academy of Health Sciences Hetauda Nepal; ^5^ School of Public Health and Community Medicine B.P. Koirala Institute of Health Sciences Dharan Nepal; ^6^ Department of Clinical Pharmacology and Therapeutics B.P. Koirala Institute of Health Sciences Dharan Nepal; ^7^ Department of Nursing Madan Bhandari Academy of Health Sciences Hetauda Nepal

**Keywords:** hypertension, medication adherence, quality of life

## Abstract

**Background:**

Good adherence to antihypertensive medication is critical for optimal blood pressure control and reducing disease‐related complications. This study aimed to assess patients’ medication adherence and health‐related quality of life (HRQoL) and explore the relationship between medication adherence and HRQoL among hypertensive patients.

**Methods:**

A cross‐sectional study was carried out at the Scheer Memorial Adventist Hospital (SMAH) in Bagmati Province, Nepal, from March 2023 to June 2023. The General Medication Adherence Scale (GMAS) and the European Quality of Life‐5 Dimensions‐5 Levels (EQ‐5D‐5L) questionnaires were used to assess medication adherence and HRQoL, respectively. Logistic regression analysis was employed to explore the relationship between participants’ characteristics, medication adherence levels, and HRQoL impairment across the five dimensions of EQ‐5D‐5L. Statistical significance was set at *p* < 0.05.

**Results:**

A total of 300 patients were surveyed, of whom only 52.33% were adherent to their antihypertensive medication. Similarly, the mean (±SD) GMAS score of the participants was 25.21 (±6.5), and the mean (SD) EQ index and EQ‐VAS scores were 0.84 ± 0.22 and 72.22 ± 11.67, respectively. The percentages of issues in various dimensions were 47.3% for mobility, 19.66% for self‐care, 26.66% for usual activities, 70.33% for pain/discomfort, and 58% for anxiety/depression. Multiple logistic regression analysis revealed that higher education attainment, longer disease duration, and enrollment to health insurance schemes were significantly associated with improve patients’ medication adherence (*p* < 0.05). Participants who were adherent to prescribed medication had lower odds of reporting problems with mobility (OR 0.21, 95% CI 0.11–0.39; *p* < 0.001), usual activities (OR 0.52, 95% CI 0.27–1.00; *p* = 0.05), pain (OR 0.17, 95% CI 0.08–0.32; *p* < 0.001), and anxiety (OR 0.36, 95% CI 021–0.62; *p* < 0.001).

**Conclusions:**

Medication adherence was suboptimal in substantial proportion of hypertensive patients. Adherence to medication was linked to an improved HRQoL in patients with hypertension regardless of sociodemographic and clinical variables.

## Graphical abstract

In a study conducted with 300 hypertensive patients in Bagmati Province, Nepal, it was found that medication adherence, which was reported at 52%, was linked to an improvement in health‐related quality of life. This enhancement was demonstrated by a decrease in problems associated with mobility, self‐care, usual activities, pain/discomfort, and anxiety/depression. These findings highlight the crucial role of adherence in enhancing patient well‐being.

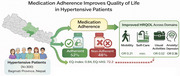

## 1 Introduction

Hypertension, commonly known as high blood pressure, has emerged as a significant global public health concern because of its rapidly rising prevalence in both industrialized and developing nations [1]. Worldwide, more than one billion individuals are affected by hypertension, which is the primary cause of mortality, leading to nearly 9.4 million deaths annually and accounting for one in every eight deaths [2]. In Nepal, hypertension and its related health issues present a significant challenge to healthcare providers, with a national prevalence rate of 25%, and most individuals with high blood pressure do not have adequate management [3]. As a result, health systems have continued to bear a tremendous clinical and financial burden owing to hypertension in recent years [4].

However, hypertension is a modifiable cardiovascular risk factor, and effective control measures significantly reduce fatal cardiovascular events and the associated morbidities and mortalities [5, 6]. A recent meta‐analysis showed that a 10 mmHg reduction in systolic blood pressure (SBP) lowered the risk of major cardiovascular events by 20%, coronary heart disease by 17%, stroke by 27%, heart failure by 28%, and death from all causes by 13% [7]. Long‐term adherence to antihypertensive medication is essential to achieve positive outcomes [8]. According to the WHO, medication adherence is “the extent to which the medication‐taking behavior of a patient corresponds with agreed recommendations from a health care provider” [9]. Despite advances in antihypertensive medication therapies, non‐adherence remains a common and significant problem, with an average adherence rate of just 50% in developed countries and an even lower rate in developing countries such as Nepal [10]. Non‐adherence to medication is prevalent in chronic conditions, such as hypertension, potentially resulting in uncontrolled blood pressure, diminished quality of life, increased healthcare expenses, and higher mortality rates. Health‐related quality of life (HRQoL) is a complex concept that encompasses physical, psychological, social, and emotional well‐being [11]. It is a widely recognized metric for evaluating the effects of chronic illnesses such as hypertension on an individual's health. Individuals with hypertension often experience lower HRQoL than those with normal blood pressure [12]. As hypertension requires lifelong management, it might worsen HRQoL; thus, assessing the impact of medication adherence on HRQoL is relevant. More recently, there has been a shift toward enhancing the quality of life and preventing disease progression over time for people with chronic illnesses, rather than solely trying to eliminate the disease or treating only its symptoms [13]. On this basis, adherence can be considered a transitional variable, whereas HRQoL is an ultimate outcome.

Several studies in Nepal independently examined medication adherence in patients with hypertension [14, 15, 16] and HRQoL [17, 18]. None of the previous studies have explored the relationship between medication adherence and HRQoL among patients with hypertension attending tertiary healthcare centers. Although international findings suggest that better medication adherence may positively influence HRQoL, the results have been inconsistent. Some studies have shown a positive link between medication adherence and HRQoL [19, 20, 21], whereas others have not reached the same conclusion [22, 23]. Therefore, it is important to further investigate how adherence to antihypertensive medication impacts HRQoL. The main goal of this study was to examine the relationship between medication adherence and HRQoL in patients with hypertension at a tertiary care hospital in Bagmati Province, Nepal. The secondary objective was to identify other factors affecting medication adherence and HRQoL in patients with hypertension.

## 2 Materials and Methods

### 2.1 Study Design, Settings, and Participants

This cross‐sectional study was conducted among hypertensive patients who visited the outpatient department (OPD) of Scheer Memorial Adventist Hospital (SMAH) in Bagmati Province, Nepal, between March 2023 and June 2023. SMAH is a referral tertiary care hospital that provides specialized health facilities to people in the Kavrepalanchok, Sindhupalchowk, Sindhuli, Ramechhap, and Dolakha districts of Bagmati Province, Nepal.

### 2.2 Study Population and Criteria

The study population included hypertensive patients, with or without comorbidities, who underwent routine follow‐up visits to the outpatient department (OPD) of the SMAH hospital. The inclusion criteria were patients previously diagnosed with hypertension with at least 1 year of clinical history, and ongoing treatment with antihypertensive medication, and those who voluntarily agreed to participate in the study. However, patients who experienced difficulties with language comprehension, patients who were critically ill or mentally incapable of responding to research questionnaires, and those who were diagnosed with contagious diseases, such as tuberculosis and coronavirus, were excluded from this study.

### 2.3 Sampling and Sample Size

Consecutive sampling methods were employed for data collection. Using Cochran's formula, a sample size of 285 was determined, considering a prevalence rate of 24.5% [3], a 95% confidence interval, and a 5% margin of error. To account for a 10% nonresponse rate, we adjusted the estimated final sample size to 314. Finally, 300 participants were included in the final analysis.

### 2.4 Data Collection

Patients diagnosed with hypertension after a routine physician consultation were selected on the basis of a review of their OPD cards and discussions with the physician during outpatient clinic hours at the hospital. The investigator approached patients who met the study criteria, explained the purpose and objectives of the study, and evaluated their eligibility. Patients who satisfied the selection criteria were invited to participate, and written informed consent was obtained from those willing to participate before data collection. Participant data were mainly gathered through self‐administration of the research questionnaire. A structured, closed‐ended questionnaire was designed to collect sociodemographic data and clinical characteristics. The self‐administered General Medication Adherence Scale (GMAS) and European Quality of Life tool (EQ‐5D‐5L and EQ‐VAS) were used to measure medication adherence and HRQoL, respectively.

### 2.5 Medication Adherence Assessment

Adherence to antihypertensive medications was measured using the translated and validated Nepali version of the Novel GMAS [24, 25]. It consists of 11 questions that assess patient non‐adherence across three domains (five questions related to patient behavior, four related to disease and pill burden, and two cost‐related measures). Patient responses were measured on a 4‐point Likert‐type scale (always, mostly, sometimes, and never). The minimum score was 0, and the maximum score was 3. The total score was categorized into two groups: non‐adherent (scores of 26 or less) and adherent (scores of 27 or higher).

### 2.6 HRQoL Assessment

HRQoL was evaluated using the Nepali version of the EQ‐5D‐5L questionnaire, provided by EuroQol [26]. The EQ‐5D‐5L allows individuals to assess their health across five areas: mobility, self‐care, usual activities, pain/discomfort, and anxiety/depression. Each area is rated on a five‐point scale from no issues (Level 1) to severe issues (Level 5). Participants also completed the visual scale (EQ‐VAS), which offers a self‐assessed health score ranging from 0 (worst imaginable health) to 100 (perfect health). Responses indicating health states from 11,111 to 55,555 in all five domains represented the best and worst possible health states, respectively. These health states were converted into a single index value, called the utility score (EQ index score), using country‐specific value sets, with values ranging from 1 (perfect health) to 0 (worse than death). As validated country‐specific value sets have not yet been developed for Nepal, the health utility values derived from the value sets of India were used to calculate the health utility index [27], considering the sociodemographic and cultural similarities between the two countries.

### 2.7 Statistical Analysis

All analyses were performed using the Statistical Package for the Social Sciences (SPSS) version 26.0. Continuous variables are presented as mean (±SD), and categorical variables are presented as frequencies and percentages. Normality of the distribution was assessed using the Shapiro–Wilk test and histogram. A chi‐square test was conducted to identify significant associations between categorical variables. All relevant covariates were retained in the multiple logistic regressions, regardless of their individual statistical significance, to control for potential confounders. Multiple binary logistic regressions were used to identify the factors associated with medication adherence adjusting for key confounders and examine the relationship between medication adherence and the presence of problems in each HRQoL domain. For the current study, we considered any impairment in either dimension of EQ‐5D‐5L as our outcome and adherence to antihypertensive medication as our exposure. The presence of a problem indicates whether any participant has issues ranging from mild to severe (score/level: 2–5) across five dimensions: mobility, self‐care, usual activities, pain/discomfort, and anxiety/depression. Non‐adherence to antihypertensive medication was defined as a score of less than 26, whereas adherence was indicated by a score of 27 or higher to classify participants into non‐adherent and adherent groups, with the initial serving as the reference group. A *p* value of less than 0.05 (95% CI) was set at *p* < 0.05.

### 2.8 Ethical Considerations

Ethical approval for the study protocol was obtained from the Institutional Review Committee of SMAH (IRC‐SMAH, Ref. no.: 2608). Subsequently, permission to proceed with the study was obtained from the selected hospital. Participation was voluntary, and written informed consent was obtained from all participants before data collection. The rights and confidentiality of patients were maintained.

## 3 Results

A total of 300 patients were enrolled in the study, with a mean (SD) age of 56.86 (±12.436 years). More than half of the participants were female (52.3%) and had an educational attainment at the secondary level or above (58.7%). Half of the participants were employed (50%), and 62.7% earned more than 30,000 NRs. per month ($217.21/month). Of the total, 39.7% were diagnosed with hypertension in the past 6–10 years, and 38.7% of individuals reported comorbidity in addition to hypertension. More than half of the participants used a single medication daily (56.7%) and were not insured by health insurance schemes (58.3%). Table 1 shows the sociodemographic characteristics of the participants.

**TABLE 1:** Sociodemographic characteristics and clinical characteristics of the patients (*N* = 300).

Variable	*M* (±SD)	*N* (%)
**Age**	56.85 (±12.436)	
Less than or equal to 60 years		178 (59.3)
More than 60 years		122 (40.7)
**Gender**		
Male		143 (47.7)
Female		157 (52.3)
**Education**		
No‐primary education		124 (41.3)
Secondary–higher		176 (58.7)
**Marital status**		
Unmarried		60 (20)
Married		240 (80)
**Employment status**		
Unemployed		55 (18.3)
Others (retired and/or farmer)		95 (31.7)
Employed		150 (50)
**Average monthly income**		
Less than 30,000		112 (37.3)
More than 30,000		188 (62.7)
**Duration of HTN since diagnosis**		
1–5 years		70 (23.3)
6–10 years		119 (39.7)
≥11 years		111 (37)
**Comorbidities**		
No		184 (61.3)
Yes		116 (38.7)
**Number of daily medications**		
Single		170 (56.7)
Two or more		130 (43.3)
**Health insurance**		
No		175 (58.3)
Yes		125 (41.7)

Abbreviations: HTN, hypertension; M (SD), mean (standard deviation); N, number of patients.

Table 2 shows the distribution of responses for GMAS. Most respondents answered “never” or “sometimes” in the GMAS measuring item questionnaires, averaging 25.21 (±6.5) out of 33 points. A total of 52.33% of participants adhered to their medications (score ≥ 27).

**TABLE 2:** Medication adherence of the participants (*N* = 300).

Construct	GMAS measuring item description	Adherence response level *n* (%)			
		Always	Mostly	Sometimes	Never
**PBNA**	Difficulty remembering medications	6 (2)	43 (14.3)	128 (42.7)	123 (41)
	Forgetting medications due to busy schedule, travel, and other events	17 (5.7)	62 (20.7)	134 (44.7)	87 (29)
	Discontinuing medications when feeling well	3 (1)	42 (14)	71 (23.7)	184 (61.3)
	Stopping taking medications due to adverse effects	1 (0.3)	64 (21.3)	109 (36.3)	126 (42)
	Stopping medications without informing a doctor	2 (0.7)	40 (13.3)	79 (26.3)	179 (59.7)
**ADPB**	Discontinuing medications due to other medicines for additional diseases	−	28 (9.4)	77 (25.7)	195 (65)
	Finding hassle to remember medications due to medication regimen complexity	4 (1.3)	59 (19.7)	83 (27.7)	154 (51.3)
	Missing medicines due to progression of disease and addition of new medicines in the last month	4 (1.3)	36 (12)	79 (26.3)	181 (60.3)
	Altering medication regimen, dose, and frequency	2 (0.7)	62 (20.7)	140 (46.7)	96 (32)
**CRNA**	Discontinuing medications because they feel that they are not worth for the money	14 (4.7)	61 (20.3)	89 (29.7)	136 (45.3)
	Find it difficult to buy medicines because they are expensive	5 (1.7)	53 (17.7)	64 (21.3)	178 (59.3)
**Overall GMAS mean (±SD) score**		**25.21 ± 6.5**			

*Note:* Always , 0; mostly , 1; sometimes , 2; never , 3.

Abbreviations: ADPB, additional disease and pill burden; CRNA, cost‐related non‐adherence; GMAS, General Medication Adherence Scale; PBNA, patient behavior‐related non‐adherence.

Predictor variables for medication adherence were identified using multiple logistic regression analysis. Educational attainments, duration of hypertension since diagnosis, comorbidities, and health insurance were found to be significantly associated with medication adherence. Patients with secondary to higher education were more likely to be adherent to antihypertensive medications (OR 2.73; 95% CI: 1.49–5.004; *p* = 0.001). Patients with longer history of hypertension diagnosis demonstrate a higher likelihood of adherence compared to those with shorter duration of diagnosis. Furthermore, patients on health insurance (OR 2.335; 95% CI 1.38–3.951; *p* = 0.002) were more likely to be adherent to medication as compared to those who were uninsured. In contrast, patients with comorbidities (OR 0.446; 95% CI 0.239–0.835; *p* = 0.012) were less likely to be adherent to medication as compared to those without comorbidities (Table 3).

**TABLE 3:** Factors associated with medication adherence in hypertensive patients.

	Medication adherence		COR	AOR	
Variables	Non‐adherent (%)	Adherent (%)	(95% CI)	(95% CI)	*p* value
**Age**					
Less than or equal to 60 years	81 (45.51)	97 (54.49)	1 (Ref.)	1 (Ref.)	0.607
More than 60 years	62 (50.82)	60 (49.18)	0.81 (0.509–1.282)	0.82 (0.38–1.76)	
**Gender**					
Male	70 (48.95)	73 (51.05)	1 (Ref.)	1 (Ref.)	0.594
Female	73 (46.50)	84 (53.50)	1.10 (0.70–1.73)	1.15 (0.68–1.94)	
**Education**					
No‐primary education	77 (62.10)	47 (37.90)	1 (Ref.)	1 (Ref.)	0.001
Secondary to higher	66 (37.50)	110 (62.50)	2.73 (1.70–4.38)	2.73 (1.49–5.00)	
**Marital status**					
Unmarried	27 (45.00)	33 (55.00)	1 (Ref.)	1 (Ref.)	0.308
Married	116 (48.33)	124 (51.67)	0.88 (0.49–1.54)	0.69 (0.34–1.41)	
**Employment status**					
Unemployed	37 (67.27)	18 (32.73)	1 (Ref.)	1 (Ref.)	0.718
Others (retired and/or farmers)	44 (46.32)	51 (53.68)	2.38 (1.19–4.76)	1.42 (0.59–3.42)	0.43
Employed	62 (41.33)	88 (58.67)	2.92 (1.52–5.59)	1.27 (0.41–3.96)	0.683
**Income per month**					
Less than 30,000	67 (59.82)	45 (40.18)	1 (Ref.)	1 (Ref.)	0.389
More than 30,000	76 (56.72)	112 (59.57)	2.19 (1.36–3.53)	1.39 (0.66–2.92)	
**Duration of HTN since diagnosis**					
1–5 years	42 (60.00)	28 (40.00)	1 (Ref.)	1 (Ref.)	<0.001
6–10 years	57 (47.90)	62 (52.10)	1.63 (0.89–2.96)	2.11 (1.03–4.33)	0.043
More than 11 years	44 (39.64)	67 (60.36)	2.28 (1.24–4.20)	6.69 (2.74–16.33)	<0.001
**Comorbidities**					
No	72 (39.13)	112 (60.87)	1 (Ref.)	1 (Ref.)	0.012
Yes	71 (61.21)	45 (38.79)	0.41 (0.25–0.65)	0.45 (0.24–0.84)	
**Number of daily medications**					
Single	73 (42.94)	97 (57.06)	1 (Ref.)	1 (Ref.)	0.27
Two or more	70 (53.85)	60 (46.15)	0.65 (0.40–1.02)	0.69 (0.36–1.33)	
**Health insurance**					
No	100 (57.14)	75 (42.86)	1 (Ref.)	1 (Ref.)	0.002
Yes	43 (34.40)	82 (65.60)	2.54 (1.58–4.08)	2.34 (1.38–3.95)	

Abbreviations: AOR, adjusted odds ratio; CI, confidence interval; COR, crude odds ratio; HTN, hypertension.

The mean (SD) health utility score (EQ index) and the EQ‐VAS scores were 0.84 ± 0.22 and 72.22 ± 11.67, respectively. The dimension with the highest prevalence of problems was pain/discomfort (70.33%), followed by anxiety/depression (58%) and mobility (47.3%), whereas self‐care problems were the least reported (19.66%). The proportions of the five dimensions of the five EQ‐5D levels and mean (SD) for EQ index and EQ‐VAS are presented in Table 4.

**TABLE 4:** HRQoL of patients by EQ‐5D‐5L dimensions (*N* = 300).

	Responses (*n*)				
EQ‐5D dimensions	No problem	Slight problems	Moderate problems	Severe problems	Extreme problems
Mobility	158	96	33	13	0
Self‐care	241	51	8	0	0
Usual activities	220	67	10	3	0
Pain/Discomfort	89	145	54	8	4
Anxiety/Depression	126	128	26	20	0
	Mean	SD			
EQ‐5D index	0.84	0.22			
EQ‐VAS	72.22	11.67			

*Note:* Data are expressed as frequency (*n*), and EQ‐5D‐5L dimensions were coded as no problem = 1; slight problems = 2; moderate problems = 3; severe problems = 4; and extreme problems = 5. Mean (SD) values for EQ‐5D index and EQ‐VAS were presented.

Abbreviation: HRQoL, health‐related quality of life.

Table 5 presents the results of the univariate analysis of HRQoL. Medication non‐adherence was significantly associated with a higher proportion of patients reporting problems in all EQ‐5D‐5L domains. The univariate effects of the other variables varied from those of the dependent variable.

**TABLE 5:** Univariate analysis of factors associated with the presence of problems in each domain of EQ‐5D.

	Mobility problems		Self‐care problems		Usual activities problems		Pain/Discomfort problems		Anxiety/Depression problems	
Factors	*N* (%)	*p* value	*N* (%)	*p* value	*N* (%)	*p* value	*N* (%)	*p* value	*N* (%)	*p* value
**Age**										
Less than or equal to 60 years	56 (31.46)	<0.001	13 (7.30)	<0.001	30 (16.85)	<0.001	110 (61.79)	<0.001	102 (57.30)	0.768
More than 60 years	86 (70.49)		46 (37.70)		50 (40.98)		101 (82.79)		72 (59.02)	
**Gender**										
Male	64 (44.76)	0.393	32 (22.38)	0.26	39 (27.27)	0.821	99 (69.23)	0.69	83 (58.04)	0.989
Female	78 (49.68)		27 (17.20)		41 (26.11)		112 (71.34)		91 (57.96)	
**Education**										
No‐primary education	78 (62.90)	<0.001	41 (33.06)	<0.001	49 (39.52)	<0.001	100 (80.65)	0.001	85 (68.55)	0.002
Secondary to higher	64 (36.36)		18 (10.23)		31 (17.61)		111 (63.07)		89 (50.57)	
**Marital status**										
Unmarried	14 (23.33)	<0.001	5 (8.33)	0.014	8 (13.33)	0.009	36 (60)	0.05	32 (53.33)	0.413
Married	128 (53.33)		54 (22.5)		72 (30)		175 (72.92)		142 (59.17)	
**Employment status**										
Unemployed	44 (80)	<0.001	24 (43.64)	<0.001	27 (49.1)		46 (83.64)	0.002	35 (63.64)	0.356
Others (retired and/or farmers)	56 (58.95)		27 (28.42)		31 (32.63)	<0.001	73 (76.84)		58 (61.05)	
Employed	42 (28)		8 (5.33)		22 (14.66)		92 (61.33)		81 (54)	
**Income per month**										
Less than 30,000	83 (74.11)	<0.001	40 (32.79)	<0.001	50 (44.64)	<0.001	94 (83.93)	<0.001	73 (65.18)	0.052
More than 30,000	59 (31.38)		19 (10.11)		30 (15.96)		117 (62.23)		101 (53.72)	
**Comorbidities**										
No	65 (35.32)	<0.001	24 (12.77)	<0.001	37 (20.11)	0.001	111 (60.33)	<0.001	89 (48.37)	<0.001
Yes	77 (66.38)		35 (30.17)		43 (37.07)		100 (86.21)		85 (73.28)	
**Number of daily medications**										
Single	65 (38.24)	<0.001	27 (15.88)	0.059	40 (23.53)	0.16	109 (64.12)	0.007	86 (50.59)	0.003
Two or more	77 (59.23)		32 (24.62)		40 (30.77)		102 (78.46)		88 (67.69)	
**Duration of HTN since diagnosis**										
1–5 years	25 (35.71)	0.007	10 (14.29)	<0.001	13 (18.57)	0.003	44 (62.86)	0.182	42 (60)	0.874
6–10 years	52 (43.7)		13 (10.92)		25 (21.01)		83 (69.75)		67 (56.30)	
More than 11 years	65 (58.56)		36 (32.43)		42 (37.84)		84 (75.68)		65 (58.56)	
**Health insurance**										
No	86 (49.14)	0.458	35 (20)	0.864	49 (28)	0.537	124 (70.86)	0.814	106 (60.57)	0.286
Yes	56 (44.8)		24 (19.2)		31 (24.8)		87 (69.6)		68 (54.4)	
**Mediation adherence**										
Non‐adherent	94 (65.73)	<0.001	39 (27.27)	0.002	50 (34.97)	0.002	125 (87.41)	<0.001	105 (73.43)	<0.001
Adherent	48 (30.57)		20 (12.74)		30 (19.11)		86 (54.78)		69 (43.95)	

*Note: p* value calculated using chi‐square test.

Abbreviations: GMAS, General Medication Adherence Scale; HTN, Hypertension.

The results of multivariable analyses revealed that older patients (>60 years), females, married, and those with an income of less than 30,000 NRs per month, patients with comorbidities, and non‐adherent patients were more likely to report mobility problems. Participants who earn less than 30,000 NRs per month and non‐adherent participants had a higher likelihood of reporting problems in their usual activities. A higher likelihood of reporting problems in the pain/discomfort dimensions was associated with older age (>60 years) and medication non‐adherence. An increased risk of anxiety/depression problems was associated with comorbidities and non‐adherence (Table 6).

**TABLE 6:** Multiple binary logistic regressions with the presence of problems in each EQ‐5D descriptive system's domain as the dependent variable.

	Mobility problems		Self‐care problems		Usual activities problems		Pain problems		Anxiety/Depression problems	
Factors	OR (95% CI)	*p* value	OR (95% CI)	*p* value	OR (95% CI)	*p* value	OR (95% CI)	*p* value	OR (95% CI)	*p* value
**Age**										
Less than or equal to 60 years (Ref.)										
More than 60 years	2.65 (1.19, 6.01)	0.018	2.11 (0.84, 5.44)	0.115	1.34 (0.60, 2.96)	0.472	2.39 (1.03, 5.68)	0.044	0.64 (0.31, 1.33)	0.239
**Gender**										
Male (Ref.)										
Female	1.29 (0.72, 2.35)	0.039	0.59 (0.29, 1.19)	0.148	0.83 (0.46, 1.49)	0.534	1.23 (0.69, 2.22)	0.488	1.04 (0.63, 1.74)	0.876
**Education**										
No‐primary education (Ref.)										
Secondary to higher	0.99 (0.52, 1.92)	0.099	0.64 (0.30, 1.37)	0.251	0.71 (0.37, 1.35)	0.29	0.85 (0.42, 1.71)	0.646	0.59 (0.32, 1.08)	0.087
**Marital status**										
Unmarried (Ref.)										
Married	2.66 (1.18, 6.30)	0.022	1.81 (0.60, 6.32)	0.314	1.66 (0.69, 4.34)	0.277	1.00 (0.47, 2.09)	0.993	1.15 (0.58, 2.29)	0.693
**Employment**										
Unemployed (Ref.)										
Others (retired and/or farmers)	0.689 (0.24, 1.88)	0.457	0.68 (0.28, 1.66)	0.401	0.86 (0.38, 1.98)	0.729	1.70 (0.54, 5.36)	0.363	1.53 (0.63, 3.74)	0.347
Employed	0.45 (0.13, 1.55)	0.205	0.26 (0.06, 0.98)	0.051	0.71 (0.23, 2.22)	0.56	1.86 (0.47, 7.41)	0.375	1.29 (0.42, 4.03)	0.658
**Income per month**										
Less than 30,000 (Ref.)										
More than 30,000	0.35 (0.16, 0.76)	0.008	0.62 (0.25, 1.51)	0.287	0.41 (0.19, 0.87)	0.02	0.43 (0.17, 1.01)	0.058	0.79 (0.38, 1.63)	0.524
**Comorbidities**										
No (Ref.)										
Yes	2.24 (1.11, 4.56)	0.025	1.99 (0.89, 4.55)	0.097	1.84 (0.92, 3.72)	0.086	0.79 (0.38, 1.65)	0.536	2.17 (1.16, 4.11)	0.016
Number of daily medications										
Single (Ref.)										
Two or more	1.05 (0.51, 2.14)	0.901	0.7 (0.30, 1.58)	0.396	0.61 (0.29, 1.24)	0.181	1.49 (0.69, 3.26)	0.316	1.32 (0.69, 2.50)	0.396
**Duration of hypertension since diagnosis**										
1–5 years (Ref.)										
6–10 years	1.37 (0.60, 3.19)	0.457	0.77 (0.27, 2.28)	0.631	1.26 (0.54, 3.07)	0.602	1.49 (0.69, 3.26)	0.316	0.76 (0.38, 1.53)	0.444
More than 11 years	1.38 (0.52, 3.70)	0.514	1.67 (0.54, 5.24)	0.374	2.16 (0.83, 5.79)	0.117	1.60 (0.62, 4.14)	0.332	0.81 (0.35, 1.86)	0.618
**Health insurance**										
No (Ref.)										
Yes	1.27 (0.69, 2.34)	0.442	1.56 (0.74, 3.35)	0.25	1.14 (0.62, 2.13)	0.668	1.32 (0.73, 2.42)	0.368	1.05 (0.62, 1.78)	0.853
**Mediation adherence**										
Non‐adherent (Ref.)										
Adherent	0.21 (0.11, 0.39)	<0.001	0.46 (0.21, 1.01)	0.055	0.52 (0.27, 1.00)	0.05	0.17 (0.08, 0.32)	<0.001	0.36 (0.21, 0.62)	<0.001

*Note:* For EQ‐5D‐5L, we consider the “problem” if mild to severe or an inability to perform any work (score/level: 2–5) in the case of mobility, self‐care, usual activities, pain/discomfort, and anxiety depression.

## 4 Discussion

This single‐center, cross‐sectional, and non‐interventional study aimed to evaluate medication adherence, HRQoL, and the association between medication adherence and HRQoL among hypertensive patients in the Bagmati Province of Nepal. Patients with chronic conditions, such as hypertension, are likely to experience suboptimal physical health, emotional challenges, and social limitations compared to those without chronic illnesses [28]. This study demonstrated a significant association between medication adherence and the quality of life of hypertensive patients. Adherent participants had lower odds of reporting mobility problems, usual activity problems, pain problems, and anxiety/depression problems among HRQoL dimensions compared to non‐adherent participants, indicating that consistent medication use contributes to better physical and psychological well‐being. These findings are consistent with the previous studies that have demonstrated positive relations with adherence and quality of life among hypertensive patients [20, 29, 30]. Findings from these studies indicated that adherent participants were more likely to have higher perceptions of quality of life and health, especially in the physical and psychological domains, than their non‐adherent counterparts [20]. These similarities suggest that medication adherence is a universal determinant of HRQoL, regardless of cultural or healthcare system discrepancies.

Non‐adherence to medication not only contributes to disease‐related complications but is also associated with increased hospital admissions and higher healthcare costs [4]. In our study, 36.3% of patients indicated that they occasionally discontinued their medication due to the side effects they encountered. Furthermore, 20.3% and 17.7% of the participants ceased taking their medication because they either did not believe it was worth the expense or found it financially burdensome given the high cost of medicines. Similarly, a study from Dharan, Eastern Nepal, revealed that 55.1% of patients forgot to take their medications [31], a result comparable to our study's finding of 42.7%. Factors, such as the complexity of medications, use of alternative treatments, fear of dependency, and negative side effects, have been recognized as possible obstacles to medication adherence [31, 32, 33]. Previous studies from South Asia, including Nepal, have reported medication adherence rates ranging from 32% to 95%, with variations depending on regional and cultural differences in healthcare delivery systems [34]. Our study reported a suboptimal level of adherence to antihypertensive medication at a rate of 52.33%. This finding is consistent with the previous studies conducted in Nepal (51.9%), Laos (50%), and Malaysia (53.4%) [14, 35, 36]. However, it was lower than that reported in other studies in Nepal and Bangladesh [15, 37]. We found that adherence was significantly associated with educational attainment of patients. Patients with secondary to higher education were more likely to be adherent than patients with no or primary education. This finding agrees with the previous studies [38, 39]. This may be due to the influence of individual levels of education on health literacy and their capacity to understand health information and make informed decisions about their well‐being. The participants’ educational background allows them to understand the need of adhering to medication and properly follow their treatment regimens [40].

Duration of hypertension since diagnosis was also found to affect adherence. Participants with a long history of hypertension were more likely to be adherent than those with a shorter history of hypertension. These findings are consistent with the previous studies [38, 41]. This pattern indicates prolonged exposure to disease and subsequent experiences to medicine management over time may have enabled patients to develop habitual medication‐taking routines to promote adherence. However, it is also plausible that patients with longer disease duration have had more frequent healthcare interactions, reinforcing adherence through repeated counseling and follow‐up. Conversely, participants with comorbidities were less likely to be adherent to medications, as in line with previous evidence [42]. This may be attributed to the increased complexity of treatment regimens and the burden of polypharmacy among patients with multiple comorbid conditions. Medication regimen complexity can lead to confusion and risk of medication mismanagement that pose hurdles to maintaining strict adherence. This finding highlights the need for simplified treatment strategies and targeted adherence support for multimorbid patients. Furthermore, health insurance coverage emerged as a significant positive predictor of adherence, which is consistent with findings from previous studies, including Nepal [16, 43]. This finding underscores the importance of financial accessibility in patients with chronic disease. Health insurance could minimize out‐of‐pocket expenditures, improve medication affordability, and ensure continuity of care, thereby facilitating better medication adherence.

In the current study, we found that all participants had some degree of impairment in every dimension of the EQ‐5D‐5L, indicating that none of them were entirely free from health‐related limitations. This suggests that hypertension adversely affects multiple aspects of daily life and well‐being. The findings from a previous study conducted in Nepal also reported similar problems, where most participants exhibited a moderate quality of life score with problems across all dimensions of HRQoL, and factors, such as sociodemographics, compliance with dietary salt, and self‐perceived health status, were associated with influencing HRQoL [17].

Participants aged ≥60 years were more likely to report problems related to mobility and pain. This finding is consistent with previous studies that suggest that aging is often accompanied by increased physical and psychological challenges, including mobility limitations, falls, accidents, and decreased cognitive functions, all of which collectively contribute to a reduced quality of life [44, 45].

Compared to the low‐income group, individuals in the high‐income group were significantly associated with improved HRQoL, with lower odds of having mobility problems and usual activity problems. This finding is consistent with previous studies that have reported that individuals with high‐income levels exhibit better physical health, psychological health, and social functioning [46, 47]. This could be attributed to enhanced access to healthcare resources, follow‐up, medication affordability, and adherence to therapy because of the high socioeconomic status.

The presence of multiple comorbid conditions can worsen the impact on the quality of life. Our study found that patients with comorbidities were significantly more likely to experience mobility and emotional/psychological issues than those without such conditions. This aligns with previous findings [47, 48]. These results emphasize the importance of a holistic approach that focuses on preventing and managing multiple health conditions rather than just treating individual diseases.

However, our study has certain limitations that should be considered when interpreting the findings. First, the study finding may have been affected with higher proportion of patients with treatment‐resistant hypertension who attended a hospital during the study period. As these individuals may differ from the broader hypertensive population in terms of disease severity and healthcare‐seeking behavior, the possibility of this selection bias cannot be excluded. Second, as a cross‐sectional survey relying on self‐reported data only, we did not collect objective clinical measurements such as blood pressure levels, renal function tests, or electrolyte values. These factors limit the ability to fully characterize the clinical status of participants and should be considered when interpreting the results. Third, this study did not employ a new‐user design and instead included prior diagnosed hypertensive patients who were already under antihypertensive treatment. This may have led to an overrepresentation of individuals who continued treatment, possibly because they experienced better tolerance or maintained a satisfactory quality of life. In contrast, those with poor adherence or unfavorable experiences may have been underrepresented. This could result in an overestimation of medication adherence, and the findings should be interpreted cautiously, particularly for patients newly starting treatment or experiencing difficulties with therapy. Fourth, the study was conducted at a single tertiary care center, which may limit the generalizability of the findings to other settings or populations. In addition, the use of self‐reported measures introduces the potential for recall bias, which could have affected the accuracy of reported medication adherence and its association with HRQoL. Finally, the cross‐sectional nature of the study also limited our ability to follow participants over time and establish a causal link between medication adherence and HRQoL, such as whether high adherence leads to better HRQoL or vice versa. Furthermore, a generic HRQoL measure was used, which might not be as sensitive as the disease‐specific measures.

Besides, this study has important clinical implications; the low medication adherence observed among participants with hypertension is concerning, and the negative association between adherence and HRQoL dimensions suggests that promoting adherence should be a key component of routine hypertension care. Clinicians should evaluate medication adherence at every follow‐up visit to identify non‐adherence and enable timely interventions to improve patients’ HRQoL. Although the findings suggest an association between medication adherence and HRQoL, the cross‐sectional design of the study does not allow for causal inferences. Further longitudinal studies are needed to explore whether medication adherence leads to improvements in HRQoL over time.

## 5 Conclusion

The research found that medication adherence was suboptimal in substantial proportion of hypertensive patients. Medication adherence was positively associated with higher education, longer hypertension duration, and health insurance enrollment. Furthermore, the findings highlight significant patient‐reported HRQoL problems that indicate substantial disease burden beyond clinical measures on these patients. Notably, medication adherence was found to be an independent predictor of HRQoL. Patients who adhere to their medication regimens tend to have a higher quality of life than those who do not. Further studies should explore obstacles to medication adherence and in broader contexts to enhance patient healthcare outcomes.

### 5.1 Recommendation to Policy and Practices

These findings emphasize the urgent need for targeted interventions to improve adherence and call for expansion of healthcare and insurance programs to improve patient‐centered health outcomes in hypertension management in this region. Local authorities are encouraged to create health education programs and community‐based healthcare interventions to raise disease awareness and improve medication management in individuals with chronic conditions. Efforts to decrease the number of pills and promote participation in the National Health Insurance program for those with chronic illnesses, such as hypertension, should be supported. Healthcare providers are advised to focus on patient‐centered and personalized treatment plans to boost medication adherence and HRQoL. Moreover, action should be taken at all levels to increase patient access to medical services and drug therapies, thereby enhancing the quality of life of individuals with chronic diseases.

## Author Contributions

**Supuspa Lama**: conceptualization, investigation, writing – original draft, methodology, writing – review and editing, formal analysis, project administration, data curation. **Prasanna Dahal**: conceptualization, methodology, validation, writing – review and editing, supervision, data curation. **Sunil Neupane**: conceptualization, writing – original draft, investigation, project administration, writing – review and editing. **Bipindra Pandey**: methodology, validation, writing – review and editing, data curation, project administration. **Sagun Kharel**: investigation, project administration, formal analysis, data curation, writing – review and editing. **Kadir Alam**: methodology, validation, visualization, formal analysis, supervision, data curation. **Dharanidhar Baral**: methodology, visualization, data curation, formal analysis.

## Funding

The authors have nothing to report.

## Conflicts of Interest

The authors declare no conflicts of interest.

## Data Availability

The datasets generated and/or analyzed during the current study are available from the corresponding author upon reasonable request.

## References

[1] S. Bromfield and P. Muntner , “High Blood Pressure: The Leading Global Burden of Disease Risk Factor and the Need for Worldwide Prevention Programs,” Current Hypertension Reports 15, no. 3 (June 2013): 134–136, 10.1007/s11906-013-0340-9. 23536128 PMC3699411

[2] World Health Organization ,A Global Brief on Hypertension: Silent Killer, Global Public Health Crisis: World Health Day 2013 (WHO, 2013), www.who.int.

[3] B. Bista , M. Dhimal , S. Bhattarai , et al., “Prevalence of Non‐Communicable Diseases Risk Factors and Their Determinants: Results From STEPS Survey 2019, Nepal,” PLoS One 16, no. 7 (2021): e0253605, 10.1371/journal.pone.0253605. 34329300 PMC8323895

[4] S. Lamichhane , A. Pokhrel , and N. R. Sharma , “Medication Non‐Compliance: A Challenge in Treating Hypertension in Nepal,” Annals of Medicine and Surgery 81 (2022): 104362, 10.1016/j.amsu.2022.104362. 36147085 PMC9486534

[5] P. Verdecchia , J. A. Staessen , F. Angeli , et al., “Usual Versus Tight Control of Systolic Blood Pressure in Non‐Diabetic Patients With Hypertension (Cardio‐Sis): An Open‐Label Randomised Trial,” Lancet 374, no. 9689 (August 2009): 525–533, 10.1016/S0140-6736(09)61340-4. 19683638

[6] J. A. Staessen , R. Fagard , L. Thijs , et al., “Randomised Double‐Blind Comparison of Placebo and Active Treatment for Older Patients With Isolated Systolic Hypertension,” Lancet 350 (1997): 757–764. 9297994 10.1016/s0140-6736(97)05381-6

[7] D. Ettehad , C. A. Emdin , A. Kiran , et al., “Blood Pressure Lowering for Prevention of Cardiovascular Disease and Death: A Systematic Review and Meta‐Analysis,” Lancet 387, no. 10022 (March 2016): 957–967, 10.1016/S0140-6736(15)01225-8. 26724178

[8] M. Krousel‐Wood , S. Thomas , P. Muntner , and D. Morisky , “Medication Adherence: A Key Factor in Achieving Blood Pressure Control and Good Clinical Outcomes in Hypertensive Patients,” Current Opinion in Cardiology 19, no. 4 (July 2004): 357–362, 10.1097/01.hco.0000126978.03828.9e. 15218396

[9] E. Sabaté and World Health Organization , Adherence to Long‐Term Therapies: Evidence for Action (World Health Organization, 2003).

[10] P. Pokharel , S. K. Jha , A. Adhikari , et al., “Non‐Adherence to Anti‐Hypertensive Medications in a Low‐Resource Country Nepal: A Systematic Review and Meta‐Analysis,” Annals of Medicine and Surgery 85, no. 9 (July 2023): 4520–4530, 10.1097/MS9.0000000000001088. 37663734 PMC10473346

[11] M. A. Testa and D. C. Simonson , “Assessment of Quality‐of‐Life Outcomes,” New England Journal of Medicine 334, no. 13 (March 1996): 835–840, 10.1056/NEJM199603283341306. 8596551

[12] D. J. Trevisol , L. B. Moreira , A. Kerkhoff , S. C. Fuchs , and F. D. Fuchs , “Health‐Related Quality of Life and Hypertension: A Systematic Review and Meta‐Analysis of Observational Studies,” Journal of Hypertension 29, no. 2 (February 2011): 179–188, 10.1097/HJH.0b013e328340d76f. 21045726

[13] L. Van Wilder , E. Clays , B. Devleesschauwer , et al., “Health‐Related Quality of Life in Patients With Non‐Communicable Disease: Study Protocol of a Cross‐Sectional Survey,” BMJ Open 10, no. 9 (September 2020): e037131, 10.1136/bmjopen-2020-037131. PMC7485234 32912984

[14] B. Shrestha , Z. Ferdoush , F. Rabbi , and A. Hossain , “Adherence to Medications Among Nepali Hypertensive Population: A Hospital‐Based Cross‐Sectional Study,” Biomedical Research and Clinical Practice 3, no. 1 (2018): 1–4, 10.15761/brcp.1000159.

[15] L. Bhusal , B. Deep Pathak , B. Dhakal , et al., “Determination of Level of Self‐Reported Adherence of Antihypertensive Drug(s) and Its Associated Factors Among Patient With Hypertension at a Tertiary Care Center,” Journal of Clinical Hypertension 24, no. 11 (2022): 1444–1450, 10.1111/jch.14592. 36300812 PMC9659866

[16] T. Roka and M. Ghimire , “Medication Adherence Among Hypertensive Patients Attending a Tertiary Care Hospital in Nepal,” Journal of Nepal Health Research Council 17, no. 4 (January 2020): 521–527, 10.33314/jnhrc.v17i4.2337. 32001860

[17] S. Ghimire , P. Pradhananga , B. K. Baral , and N. Shrestha , “Factors Associated With Health‐Related Quality of Life Among Hypertensive Patients in Kathmandu, Nepal,” Frontiers in Cardiovascular Medicine 4 (November 2017): 69, 10.3389/fcvm.2017.00069. 29164136 PMC5681715

[18] N. Bhandari , B. R. Bhusal , K. C. Takma , and I. Lawot , “Quality of Life of Patient With Hypertension in Kathmandu,” International Journal of Nursing Sciences 3, no. 4 (December 2016): 379–384, 10.1016/j.ijnss.2016.10.002.

[19] N. H. Park , M. S. Song , S. Y. Shin , J. J. hye , and H. Y. Lee , “The Effects of Medication Adherence and Health Literacy on Health‐Related Quality of Life in Older People With Hypertension,” International Journal of Older People Nursing 13, no. 3 (September 2018): e12196, 10.1111/opn.12196. 29665241

[20] S. M. Khayyat , M. M. A. Mohamed , S. M. S. Khayyat , et al., “Association Between Medication Adherence and Quality of Life of Patients With Diabetes and Hypertension Attending Primary Care Clinics: A Cross‐Sectional Survey,” Quality of Life Research 28, no. 4 (April 2019): 1053–10561, 10.1007/s11136-018-2060-8. 30470970

[21] S. Jneid , H. Jabbour , A. Hajj , et al., “Quality of Life and Its Association with Treatment Satisfaction, Adherence to Medication, and Trust in Physician among Patients With Hypertension,” Journal of Cardiovascular Pharmacology and Therapeutics 23, no. 6 (November 2018): 532–542, 10.1177/1074248418784292. 29916266

[22] Y. S. Alsaqabi and U. Rabbani , “Medication Adherence and Its Association with Quality of Life among Hypertensive Patients Attending Primary Health Care Centers in Saudi Arabia,” Cureus 12, no. 12 (December 2020): e11853, 10.7759/cureus.11853. 33282607 PMC7714734

[23] F. Saleem , M. A. Hassali , A. A. Shafie , et al., “Does Treatment Adherence Correlates With Health Related Quality of Life? Findings From a Cross Sectional Study,” BMC Public Health [Electronic Resource] 12, no. 1 (December 2012): 318, 10.1186/1471-2458-12-318. 22545950 PMC3488479

[24] A. A. Naqvi , M. A. Hassali , M. Rizvi , et al., “Development and Validation of a Novel General Medication Adherence Scale (GMAS) for Chronic Illness Patients in Pakistan,” Frontiers in Pharmacology 9 (October 2018): 1124, 10.3389/fphar.2018.01124. 30356775 PMC6189444

[25] R. Shrestha , B. Sapkota , A. P. Khatiwada , et al., “Translation, Cultural Adaptation and Validation of General Medication Adherence Scale (GMAS) Into the Nepalese Language,” Patient Preference and Adherence 15 (2021): 1873–1885, 10.2147/PPA.S320866. 34475753 PMC8407778

[26] M. Herdman , C. Gudex , A. Lloyd , et al., “Development and Preliminary Testing of the New Five‐Level Version of EQ‐5D (EQ‐5D‐5L),” Quality of Life Research 20, no. 10 (2011): 1727–1736, 10.1007/s11136-011-9903-x. 21479777 PMC3220807

[27] G. Jyani , A. Sharma , S. Prinja , et al., “Development of an EQ‐5D Value Set for India Using an Extended Design (DEVINE) Study: The Indian 5‐Level Version EQ‐5D Value Set,” Value in Health: The Journal of the International Society for Pharmacoeconomics and Outcomes Research 25, no. 7 (July 2022): 1218–1226, 10.1016/j.jval.2021.11.1370. 35779943

[28] Y. Zhang , Z. Zhou , J. Gao , et al., “Health‐Related Quality of Life and Its Influencing Factors for Patients With Hypertension: Evidence From the Urban and Rural Areas of Shaanxi Province, China,” BMC Health Services Research [Electronic Resource] 16, no. 1 (July 2016): 277, 10.1186/s12913-016-1536-x. 27430314 PMC4950775

[29] R. Muyisa , E. Watumwa , D. Mataula , et al., “Quality of Life and Medication Adherence of Hypertensive Patients in Eastern DR Congo: A Cross‐Sectional Study,” Clinical Epidemiology and Global Health 34 (July 2025): 102083, 10.1016/j.cegh.2025.102083.

[30] I. A. Alhaddad , O. Hamoui , A. Hammoudeh , and S. Mallat , “Treatment Adherence and Quality of Life in Patients on Antihypertensive Medications in a Middle Eastern Population: Adherence,” Vascular Health and Risk Management 12 (October 2016): 407–413, 10.2147/VHRM.S105921. 27822055 PMC5089866

[31] B. Bhandari , M. Bhattarai , M. Bhandari , A. Ghimire , P. K. Pokharel , and D. E. Morisky , “Adherence to Antihypertensive Medications: Population Based Follow up in Eastern Nepal,” Journal of Nepal Health Research Council 13, no. 29 (2015): 38–42. 26411711

[32] E. A. Mamaghani , E. Hasanpoor , E. Maghsoodi , and F. Soleimani , “Barriers to Medication Adherence Among Hypertensive Patients in Deprived Rural Areas,” Ethiopian Journal of Health Sciences 30, no. 1 (January 2020): 85–94, 10.4314/ejhs.v30i1.11. 32116436 PMC7036465

[33] U. Mohammed Ndagi , “Medication Adherence and Its Associated Factors Among Hypertensive Patients in a Tertiary Health Facility in Minna, North Central Nigeria,” Archives of Clinical Hypertension 5, no 1 (May 2019): 3–7, 10.17352/ACH.000021.

[34] J. M. Akeroyd , “Adherence to Cardiovascular Medications in the South Asian Population: A Systematic Review of Current Evidence and Future Directions,” World Journal of Cardiology 7, no. 12 (2015): 938–947, 10.4330/wjc.v7.i12.938. 26730300 PMC4691821

[35] E. Takahashi , P. Vilay , K. Chanthakoummane , et al., “Adherence to Antihypertensive Medications in Rural Lao PDR: A Prospective Observational Study,” Tropical Medicine and Health 49 (2021): 88, 10.1186/s41182-021-00374-4. 34715938 PMC8556995

[36] A. Ramli , N. S. Ahmad , and T. Paraidathathu , “Medication Adherence Among Hypertensive Patients of Primary Health Clinics in Malaysia,” Patient Preference and Adherence 6 (2012): 613–622, 10.2147/PPA.S34704. 22969292 PMC3437910

[37] A. B. Sharif , S. S. A. Chowdhury , M. D. Z. Hossain , M. D. A. Hossain , A. Hossain , and H. M. Reza , “Prevalence and Determinants of Medication Adherence Among Hypertensive Patients: An Institution‐Based Cross‐Sectional Study,” PLoS One 20, no. 5 (May 2025): e0321449, 10.1371/journal.pone.0321449. 40392832 PMC12091766

[38] T. Win , S. Banharak , and W. Ruaisungnoen , “Factors Influencing Medication Adherence Among Patients With Hypertension: A Systematic Review,” Systematic Reviews in Pharmacy 12, no. 1 (2021): 526–538.

[39] N. R. Dangaura , P. Khanal , B. S. Kuikel , et al., “Impact of National Health Insurance on Medication Adherence Among Hypertensive Patients: A Hospital‐based Cross‐Sectional Study From Kailali, Nepal,” PLoS One 20, no. 9 (September 2025): e0332602, 10.1371/journal.pone.0332602. 40971371 PMC12448337

[40] A. Guo , H. Jin , J. Mao , et al., “Impact of Health Literacy and Social Support on Medication Adherence in Patients With Hypertension: A Cross‐Sectional Community‐Based Study,” BMC Cardiovascular Disorders [Electronic Resource] 23, no. 1 (December 2023): 93, 10.1186/s12872-023-03117-x. 36803662 PMC9940429

[41] J. Pan , L. Wu , H. Wang , et al., “Determinants of Hypertension Treatment Adherence Among a Chinese Population Using the Therapeutic Adherence Scale for Hypertensive Patients,” Medicine 98, no. 27 (2019): e16116, 10.1097/MD.0000000000016116. 31277112 PMC6635171

[42] L. Dhar , J. Dantas , and M. Ali , “A Systematic Review of Factors Influencing Medication Adherence to Hypertension Treatment in Developing Countries,” Open Journal of Epidemiology 07, no. 03 (2017): 211–250, 10.4236/ojepi.2017.73018.

[43] J. Yousuf , K. T. Roba , N. Ahmed , et al., “Antihypertensive Medication Adherence and Associated Factors Among Adult Hypertensive Patients at Public Hospitals in Eastern Ethiopia: A Cross‐Sectional Study,” PLoS One 20, no. 5 (May 2025): e0322655, 10.1371/journal.pone.0322655. 40435367 PMC12118979

[44] P. Maresova , E. Javanmardi , S. Barakovic , et al., “Consequences of Chronic Diseases and Other Limitations Associated With Old Age—A Scoping Review,” BMC Public Health [Electronic Resource] 19, no. 1 (December 2019): 1431, 10.1186/s12889-019-7762-5. 31675997 PMC6823935

[45] E. Zheng , J. Xu , J. Xu , et al., “Health‐Related Quality of Life and Its Influencing Factors for Elderly Patients With Hypertension: Evidence from Heilongjiang Province, China,” Frontiers in Public Health 9 (March 2021): 654822, 10.3389/fpubh.2021.654822. 33796501 PMC8007785

[46] G. Kandasamy , M. Almanasef , K. Orayj , A. M. Alshahrani , and S. M. Alahmari , “Assessing the Impact of Hypertension on Health‐Related Quality of Life: Insights From Sociodemographic, Economic, and Clinical Features Using SF‐36,” Healthcare 13, no. 7 (2025): 838, 10.3390/healthcare13070838. 40218136 PMC11988729

[47] A. Mannan , K. M. Akter , F. Akter , et al., “Association Between Comorbidity and Health‐Related Quality of Life in a Hypertensive Population: A Hospital‐Based Study in Bangladesh,” BMC Public Health [Electronic Resource] 22, no. 1 (December 2022): 181, 10.1186/s12889-022-12562-w. 35081905 PMC8793199

[48] Z. Liang , T. Zhang , T. Lin , et al., “Health‐Related Quality of Life Among Rural Men and Women With Hypertension: Assessment by the EQ‐5D‐5L in Jiangsu, China,” Quality of Life Research 28, no. 8 (August 2019): 2069–2080, 10.1007/s11136-019-02139-3. 30830645

